# Using Wake-Up Tasks for Morning Behavior Change: Development and Usability Study

**DOI:** 10.2196/39497

**Published:** 2022-09-21

**Authors:** Kyue Taek Oh, Jisu Ko, Jaemyung Shin, Minsam Ko

**Affiliations:** 1 Department of Human-Computer Interaction Hanyang University Ansan Republic of Korea; 2 Department of Applied Artificial Intelligence Hanyang University Ansan Republic of Korea; 3 Delightroom Seoul Republic of Korea

**Keywords:** health app design, morning behavior change, wake-up task, mobile alarm, productivity

## Abstract

**Background:**

Early morning behaviors between waking up and beginning daily work can develop into productive habits. However, sleep inertia limits the level of human ability immediately after waking, lowering a person’s motivation and available time for productive morning behavior.

**Objective:**

This study explores a design for morning behavior change using a wake-up task, a simple assignment the user needs to finish before alarm dismissal. Specifically, we set two research objectives: (1) exploring key factors that relate to morning behavior performance, including the use of wake-up tasks in an alarm app and (2) understanding the general practice of affecting morning behavior change by implementing wake-up tasks.

**Methods:**

We designed and implemented an apparatus that provides wake-up task alarms and facilities for squat exercises. We recruited 36 participants to perform squat exercises in the early morning using the wake-up tasks for 2 weeks. First, we conducted a generalized estimating equation (GEE) analysis for the first research objective. Next, we conducted a thematic analysis of the postsurvey answers to identify key themes about morning behavior change with the wake-up tasks for the second objective.

**Results:**

The use of wake-up tasks was significantly associated with both the completion of the target behavior (math task: *P*=.005; picture task: *P*<.001) and the elapsed time (picture task: *P*=.08); the time to alarm dismissal was significantly related to the elapsed time to completion (*P*<.001). Moreover, the theory of planned behavior (TPB) variables, common factors for behavior change, were significant, but their magnitudes and directions differed slightly from the other domains. Furthermore, the survey results reveal how the participants used the wake-up tasks and why they were effective for morning behavior performance.

**Conclusions:**

The results reveal the effectiveness of wake-up tasks in accomplishing the target morning behavior and address key factors for morning behavior change, such as (1) waking up on time, (2) escaping from sleep inertia, and (3) quickly starting the desired target behavior.

## Introduction

### Background

There has been rising interest in spending early morning time productively, with a view that early morning behaviors between waking up and the starting of daily work can develop into productive habits (called miracle morning) [1,2]. These habits improve health and quality of life [3]. For example, morning exercise is effective in increasing physical performance, such as muscular strength [4], anaerobic power [5], and endurance [6], while improving blood sugar [7] and hormone levels [8]. Moreover, early morning emotions can influence feelings throughout the day [9].

Many researchers have studied human behavior change [10]. For example, a conscious goal can induce the anticipated behavior [11]. Locke and Latham [12] discovered a linear relationship in which the appropriate difficulty of the goal produces a high level of effort and performance. Fogg [13,14] explained the initiation of the target behavior by 3 factors (motivation, ability, and trigger) and presented the behavior grid, which categorizes behavior change into 35 cases according to behavior type and maintenance degree. Prochaska et al [15,16] described 6 phases of behavior change (precontemplation, contemplation, preparation, action, maintenance, and termination). Such theories can provide valuable insights for developing intervention methods but are often insufficient to explain specific behavior changes in a health domain [17,18].

Under these behavior theories, studies have sought to develop computational supports for behavior change and persuasion [19-21]. Oinas-Kukkonen et al [21] classified persuasive system principles into 4 categories (primary task, dialog, system credibility, and social support). For example, tunneling can facilitate the target behavior by allowing the process to proceed without interruption. Fogg [19] proposed social cues for the persuasive system as a social actor. Oulasvirta et al [22] investigated smartphone usage from the perspective of habit formation. Consolvo et al [23] studied behavior change components by designing a mobile app that uses social supports to foster healthy behavior. There have also been studies on the impact of mobile tools on behavior change. For example, ReVibe [24] uses contextual information automatically collected from phones or sensors to improve momentary ecological assessments. FoodPrint [25] is a photo-based food diary that helps patients and health experts exchange knowledge and focus on collaboration goals. Costa et al [26] proposed a method for regulating emotions through haptic feedback with a smartwatch.

However, it is necessary to consider contextually unique characteristics for morning behavior change that differ from the prior studies in general contexts. For example, physical and cognitive performance decreased in the sleep inertia state. This is especially true of a person’s decision-making ability [27,28] and indicates that prior behavior change mechanisms may not work effectively in the morning. Thus, this study focuses on morning behavior change and attempts to understand design implications considering the contextual characteristics of morning hours.

We especially note that sleep inertia [29], which refers to the phenomenon that occurs after waking up in which people’s abilities are significantly lower than those under their normal condition, characterizes early morning behavior. Several studies have investigated physical and mental states when experiencing sleep inertia to understand the contextual characteristics of sleep inertia [28,30,31]. For example, it takes more time for someone experiencing sleep inertia to perform arithmetic calculations [32,33] and recognize objects [34,35]. Although sleep inertia usually vanishes naturally within some minutes of waking, its duration varies depending on the individual and context [34,35]. Efficiently completing target morning behaviors at a predictable time is essential because a prescheduled daily routine begins sooner (eg, going to work). Therefore, sleep inertia would be a critical factor in a person’s morning behavior performance.

Various studies aimed to determine how to escape sleep inertia quickly and effectively [36]. For example, McFarlane et al [37-39] explored auditory factors, such as alarm sounds, to decrease the influence of sleep inertia, and Hilditch et al [40] attempted to induce arousal using light of specific frequencies. Additionally, physical activity has been addressed as the primary countermeasure to sleep inertia. For example, Kaplan et al [41] proposed a method to reduce the sleep inertia duration and severity by performing a series of physical activity routines. Kovac et al [42] also measured the degree of sleepiness after the experimental participants performed the postwaking cycle and sprint.

Our study aligns with these studies in terms of helping escape sleep inertia. Although these studies focused on developing methods to escape from sleep inertia quickly and effectively, our study aims to help users maintain specific behaviors against sleep inertia, leading to morning behavior changes by associating them with a mobile alarm.

### Objectives

This study proposes a mechanism for morning behavior change based on wake-up tasks in a mobile alarm. A wake-up task is a simple assignment that the user needs to finish first to dismiss a ringing alarm, such as taking a picture of a particular object or solving math problems. Its effectiveness in waking up on time has been analyzed [43,44]. In this study, we designed and implemented a mobile alarm app based on a wake-up task to facilitate a target behavior in the morning. We also conducted formative research to understand morning behavior change by wake-up tasks, considering the following objectives: (1) exploring key factors that relate to morning behavior performance, including the use of wake-up tasks and (2) understanding the general practice of effecting morning behavior change by implementing wake-up tasks.

## Methods

### Apparatus

#### Design Requirements

We conducted a preliminary survey to understand the practice and intention of early morning behaviors. Our survey included several open-ended questions organized into 3 sections. The first section includes 2 open-ended questions about respondents’ usual morning behaviors and intentions toward productive morning behavior. The second section asks respondents to describe their previous efforts and strategies for productive morning behaviors. Finally, the survey asked all respondents to evaluate their past morning behavior and describe any difficulties they encountered. The survey was conducted through the internet and posted on social networking services in South Korea. The survey was answered by 40 participants (age: mean 30.32, SD 3.87 years), of which 17 were men (age: mean 31.64, SD 4.16 years) and 23 were women (age: mean 29.34, SD 3.04 years). The respondents were compensated with an electronic voucher worth approximately US $4. We conducted a thematic analysis following the guideline proposed by Braun and Clarke [45]. The transcripts of the responses were reread by 2 researchers, and they generated initial codes. Next, they examined the similarity between codes to define the final themes. The survey respondents are denoted by R1-R40.

First, the results show usual morning behaviors immediately after waking up. For example, 60% (24/40) of the respondents said they usually check their smartphones after waking up. Specifically, among respondents who said they usually check their smartphones, 41.6% (10/24) use social networking service or messenger apps, another 41.6% (10/24) check the time, and 16.6% (4/24) read the news on the internet. Other respondents (16/40, 40%) also reported other activities, including drinking water, going to the bathroom, making the bed, and stretching.

Furthermore, 87.5% (35/40) of respondents commented that they wish to perform more productive behaviors in the morning. For example, 1 respondent said, *“I can do simple exercise at least. I think it will be good for my health”* (R10). Another respondent mentioned, *“I think I can start the day in a good mood if I am more productive in that hours”* (R25). Some respondents also addressed the benefits of using early morning time for productive purposes. For example, a participant said, *“Taking time to do something in the evening is difficult. But, in the morning, as long as I get up early, I can have extra time to do meaningful behavior, such as stretching”* (R12). On the other hand, 5 respondents showed negative attitudes, mainly because they had been too busy in the morning. For example, a participant stated, *“I’m too busy getting ready for work, so I don’t have much time to do productive activities”* (R32).

Additionally, 75% (30/40) of the respondents had previously tried productive morning behavior. Targeted morning behaviors were mostly related to physical activity, such as stretching or simple exercising (eg, squats). Other activities like reading, writing, studying, meditation, and praying were also mentioned. These respondents explained their strategies for maintaining their desired behavior and getting up earlier is the most common strategy. Respondents also said they tended to put considerable effort into remembering the target behavior, such as forcing themselves to perform the behavior in a group setting. Next, several respondents said they had attempted to find suitable behaviors for the available morning time and had modified the goal based on the situation and performance. For example, some respondents thoroughly planned their morning time to ensure that the target behavior finished in time, whereas others attempted to find simple stretching exercises.

However, maintaining the target behavior every morning was challenging. Most responses were related to a lower level of awakening, with 82.5% (33/40) of respondents stating they often woke up late or stayed half-asleep excessively. There were also other awakening-related comments, such as demotivation. For example, “*When I got up in the morning, I did not want to do anything and hesitated to start exercising, although I planned it”* (R4).

#### Design Implications: Winding Up and Nudging by Wake-Up Tasks

Most failures affecting the targeted morning behavior are related to the characteristics of morning hours, such as limited time and respondents’ lowered ability due to sleep inertia. Therefore, 2 requirements for morning behavior support are proposed. First, it is critical to ensure that the user wakes up on time and recovers to the normal state as soon as possible. Second, it is necessary to create a bridge between waking up and starting the target behavior to prevent forgetting to perform it and avoid distractions.

**Figure 1:**
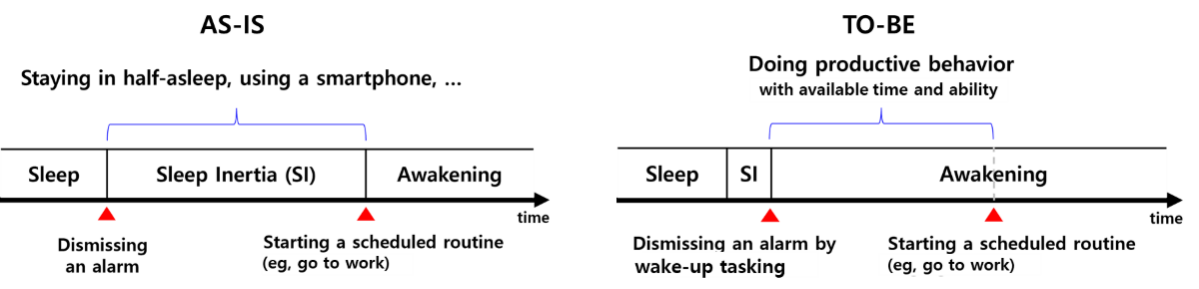
Proposed mechanism for morning behavior change by wake-up tasking.

This study proposes a new mechanism for morning behavior change by implementing a wake-up task, as shown in Figure 1. The wake-up task means a user must perform a simple task to dismiss a ringing alarm (eg, taking a picture or solving math problems) [44]. Completing a wake-up task can help users recover their normal ability quickly and have sufficient time for the target behavior. Specifically, the proposed mechanism works in two stages: (1) winding up to escape from sleep inertia by performing a wake-up task and (2) nudging to induce the target behavior after completion.

#### Implementation

**Figure 2:**
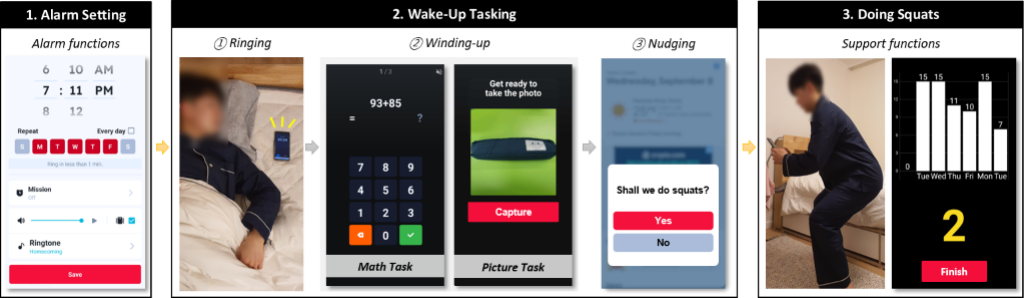
Screenshots of the study apparatus.

We implemented an alarm app that provides task-based alarm dismissal, as shown in Figure 2. The app provides three alarm dismissal methods: a conventional method as a baseline (ie, pressing a button) and two wake-up tasks (taking a picture and solving math problems). These are denoted as non_task, picture_task, and math_task, respectively. In this study, a squat exercise was selected as a target behavior. This is because simple exercising was the most preferred morning behavior, according to the preliminary study. Furthermore, doing squats is relatively easy and does not require additional equipment. The app functions as follows:

First, the app provides standard alarm functions, such as controlling the alarm sound, switching the alarm to vibrate mode, and selecting a ringtone from built-in sound sources. Notably, the user must choose an alarm dismissal method from among non_task, math_task, and picture_task. Furthermore, picture_task and math_task are representative tasks that require physical and cognitive abilities, respectively. For setting the picture_task, the user must input a reference image for later authentication. The app encourages users to select an image taken far away from their bed as the reference image. For the math_task, the user specifies the number of problems and the difficulty level. There are 6 levels of difficulty. The number of digits a user needs to calculate increases as the difficulty increases, and multiplication operations are included from level 4. The user needs to solve 3 problems by default but is allowed to change the number.

Next, when the alarm goes off, the user must complete the task required by each dismissal method; this is the winding-up stage to help escape from sleep inertia. non_task is a conventional dismissal method in many alarm apps. With non_task, the user can dismiss an alarm by simply pressing the dismissal button. math_task requires performing arithmetic calculations for the alarm dismissal, and the alarm will keep ringing until the user correctly answers the problems. With picture_task, the user must take the same picture as the registered reference image. The app determines whether the input and reference images are the same based on their image features (ie, color, shape) extracted by Android OpenCV [46]. After dismissing the alarm, the nudging stage begins with a dialog popup asking whether the user will start the target behavior immediately. If the user accepts it, the app displays functions to help the user perform the required squat exercises.

Finally, the user begins to perform the target behavior with support functions provided by the app. The users can view their performance records for the last 7 days. During the squat exercise, the app also counts the number of squat reps performed by the user and displays them on the smartphone screen. When the user performs a squat by grabbing the smartphone and looking at the screen, the app recognizes the up-and-down motion using the internal inertial measurement unit sensor values for the past 2 seconds. We built a simple deep learning model based on long short-term memory units using TensorFlow, and the trained model was converted into the TensorFlow-Lite format, which enables it to run on a mobile device. When the user finishes the exercise by pressing the “complete” button, the app asks the user to input the exact number of the reps to correct the miscounted cases. The main experiment with 36 participants discovered no significant difference between the automatically counted number of squats and the number the user inputted (auto: mean 13.5, SD 6.8; manual: mean 14.6, SD 4.4). Their Pearson correlation coefficient, 𝑟=0.636, and the mean absolute error (MAE) was 2.65, indicating 2 or 3 miscounted reps. This tendency became stronger when 3 users who had consistently reported frequent miscounts from the beginning (possibly due to device or operating system issues) were excluded (MAE=1.99, 𝑟=0.717).

### Participants

A between-group study asked users to try to do squat exercises with the apparatus in the early morning for 2 weeks. The target behavior for this study was performing 15 squats 10 minutes after the target time was set for the morning alarm. We employed generalized estimating equation (GEE) modeling in this study to measure continuous and repetitive behavioral changes. We investigated previous studies that used the GEE model as a statistical method and confirmed the sample size of this study by referring to the experimental configuration of previous studies [47-49]. Therefore, we recruited 36 participants (21 males and 15 females) who can use mobile alarms and perform squats every morning through social networking service posting. The average age of these users was 27.5 years (SD 7.68 years), and they were mostly office workers (20/36, 55.6%) and students (16/36, 44.4%). We denote these participants by P1-P36.

### Procedure

First, the participants were asked to respond to a presurvey. The presurvey asked about their demographics and usual wake-up time on a weekday. Notably, the survey included the theory of planned behavior (TPB) [50], which consists of 20 items on a 7-point Likert scale to assess traditional behavior factors (ie, intention, attitude, subjective norm, and control). Next, participants were divided into 3 groups by assigning individuals to 1 of the dismissal methods (non_task: 12, picture_task: 12, and math_task: 12). We confirmed that all variables measured by the presurvey (ie, age, gender, and TPB score) did not significantly differ among the groups. The detailed results of the presurvey are described in Multimedia Appendix 1.

Next, the 2-week experiment began, with each participant instructed to set a morning alarm using the implemented app with the assigned dismissal method. Participants were encouraged to set their alarms 5 minutes earlier to improve morning behavior. Each alarm was individually checked to ensure that it worked as intended; all users were using the dismissal method, and they performed their squat exercises with the app’s counter. Finally, participants were asked to perform 15 squats every weekday morning for 2 weeks voluntarily. We emphasized that they could skip or reject the behavior of their choosing, and we assured them that they would receive the same compensation regardless of their performance.

A postsurvey was conducted once the experiment was completed to understand detailed contexts about early morning behavior with wake-up tasks. This survey first inquired about their general usage and experience of the app, including helpful functions and failure cases. The next section of the survey questioned their alarm settings and measured the workloads of dismissing an alarm early in the morning by the NASA-Task Load Index (NASA-TLX) questionnaire [51]. We used the original NASA-TLX questionnaire in a graphic form that consists of rating bars [51]. The rating bar provides effective assistance for experimental respondents to understand and respond to the questionnaire intuitively, so we judged that the influence of linguistic factors on the experimental results was insignificant. Finally, the survey asked users to explain changes in their daily lives after the experiment.

### Analysis Methods

#### Quantifying Morning Behavior Performance

Behavior performance was quantified from two perspectives: (1) success rate and (2) time elapsed for users to start their first squat in a successful trial. The target behavior of the day was regarded as a success if the user completed 15 squats. This was denoted as a binomial variable, success, which had a value of 1 when it was a success and 0 otherwise. If the user completed the squats 15 times within 10 minutes after the morning alarm fired, the case was called early_success (note that success is a variable that includes early_success).

Unlike other domains of behavior change, early starts can be important in the morning hours because frequent morning delays cause failures by running out of available morning time. As a result, the target behavior was evaluated by measuring the elapsed time to starting the first squat in a successful trial after the alarm rang (ET_ring2success). The time between alarm ringing and dismissal (ET_ring2dismiss) was also measured, as was the time between when users dismissed the alarm and when they started their first squat in a successful trial (ET_dismiss2success).

#### Exploring Factors Affecting Morning Behavior Performance

Next, a GEE [52] analysis was performed to determine whether and how the wake-up task and alarm usage affected morning behavior performance. This study used repeated measures (eg, success, elapsed time) for the same participant. Therefore, the GEE method, widely used to handle repetitive measurements or for clustering data, was adopted [53,54]. The GEE analysis is also suitable for this study (compared with other repeated-measures analyses, including repeated-measures ANOVA) because it can use more than 2 diverse predictors. TPB behavior factors could explain behavior change better by cooperating with the alarm and wake-up task usage. Specifically, 2 dependent variables related to the performance were used, namely early_success and ET_ring2success. According to the variable type, a binomial GEE model was developed using the logit link function for early_success and another with Gaussian estimation for ET_ring2success. In total, there were 9 independent variables. The 3 groups representing the dismissal methods were represented by 2 dummy variables (eg, picture_task=1 and others=0; math_task=1 and others=0). Two variables related to alarm usage were considered: the time the alarm fires (ringing_time) and ET_ring2dismiss. Then, four subscales in the TPB questionnaire were used: (1) intention (TPB_intention), (2) attitude (TPB_attitude), (3) subjective norm (TPB_subjective_norm), and (4) control (TPB_control). Finally, we used the elapsed days (ET_days) as the last independent variable to handle repeatedly measured data.

#### Analyzing the Practice of Early Morning Behavior With Wake-Up Tasks

Finally, we aimed to understand the practice of early morning behavior with wake-up tasks based on the app’s usage logs and postsurvey responses. We conducted both quantitative and qualitative analyses. First, the NASA-TLX score on wake-up tasks was aggregated by following its original guideline [**51**], which determines weights for workload dimensions and calculates their weighted average. We conducted a 1-way ANOVA test to compare the NASA-TLX scores among the 3 groups, and a Tukey Honestly Significant Difference test was performed as the posthoc test. Second, similar to the preliminary study, 2 researchers collaboratively performed thematic analysis on postsurvey responses.

### Ethical Considerations

All subjects participated voluntarily and received US $43 each as compensation. All the study data are deidentified. This study has received an exemption from the Institutional Review Board of the Hanyang University (HYUIRB-202205-011).

## Results

### User Statistics

#### Success Rate

**Figure 3:**
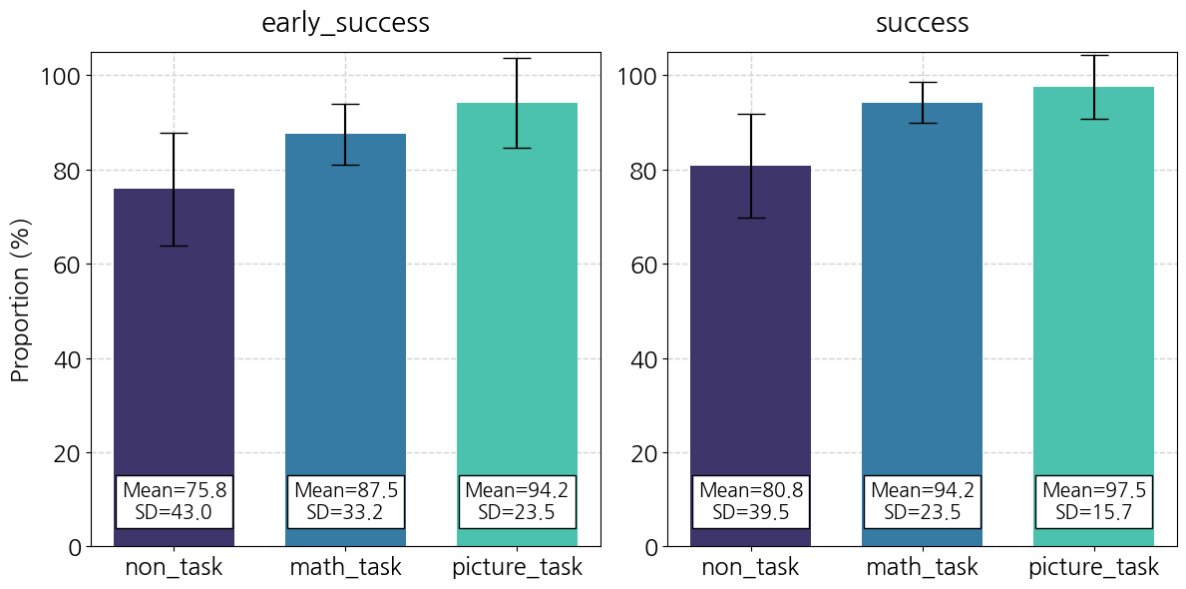
Average success rate. SD: standard deviation.

Figure 3 depicts the success rate. The picture_task group achieved the highest early_success rate (94.2%), followed by the math_task group (87.5%). This indicates that these user groups maintained the target behavior almost every weekday for 2 weeks. However, the participants in the non_task group, which used the conventional dismissal method, finished their daily squats mission only 75.8% of the time during the same period. A similar tendency occurred when adding successes to the quantity. The picture_task group (97.5%) and math_task group (94.2%) showed a success rate higher than 90% when the time limit was ignored.

**Figure 4:**
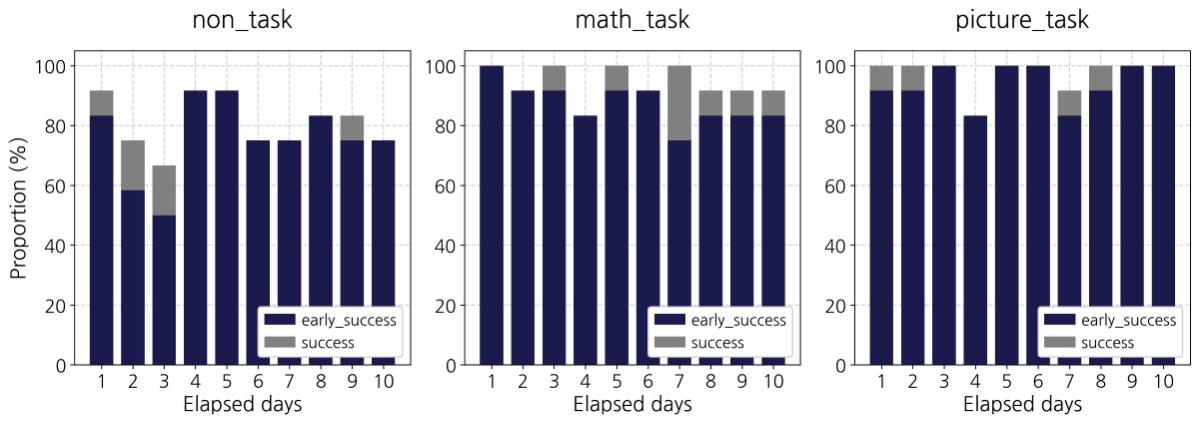
Daily success rates.

The non_task group’s success rate also increased with successes, but it remained the lowest (80.8%). As shown in Figure 4, we could not find a clear tendency over time for all groups. However, the non_task (SD 11.64) group showed a relatively higher variability in the daily success rate than the picture_task (SD 6.72) group and the math_task (SD 6.01) group.

#### Elapsed Time to Start the First Squat in a Successful Trial

**Figure 5:**
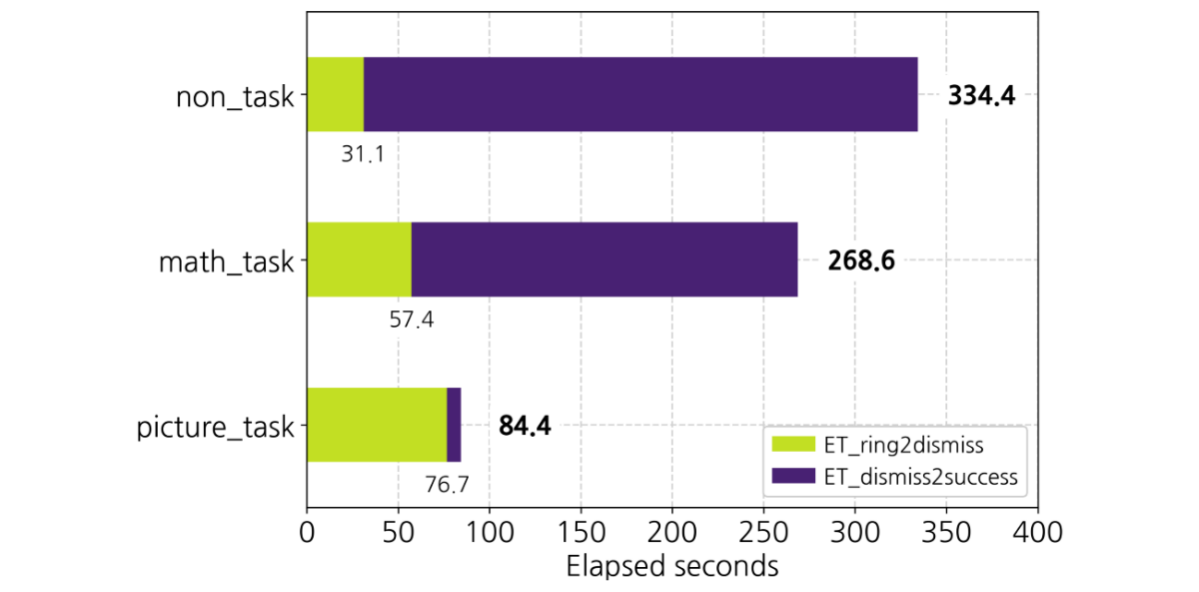
Average elapsed seconds to begin (in successful trials).

Figure 5 illustrates the timeline from alarm ringing to when the users started their first squat in a successful trial for each user group. Note that the figure presents only the trials in which the user successfully performed squats at least 15 times. We discovered that the picture_task group had the shortest time to success after the alarm rang (84.4 seconds). The math_task group (268.6 seconds) came in second, followed by the non_task group (334.4 seconds). The math_task and picture_task groups took 19-45 seconds longer than the non_task group to dismiss their alarms. This is probably because the user was required to move to a specified location to capture the preregistered image or perform the given arithmetic operations.

Nevertheless, the elapsed time to start the first squat in a successful trial tended to be much shorter in the math_task and picture_task groups than in the non_task group. In particular, the picture_task group started squats in a very short time of 7.7 seconds after dismissing the alarm. On the other hand, the non_task group’s ET_dismiss2success time is relatively longer than that of the other groups, indicating that the non_task group often delayed the target behavior after dismissing the alarm.

### Outcome 1: Factors Affecting Morning Behavior Performance

**Figure 6:**
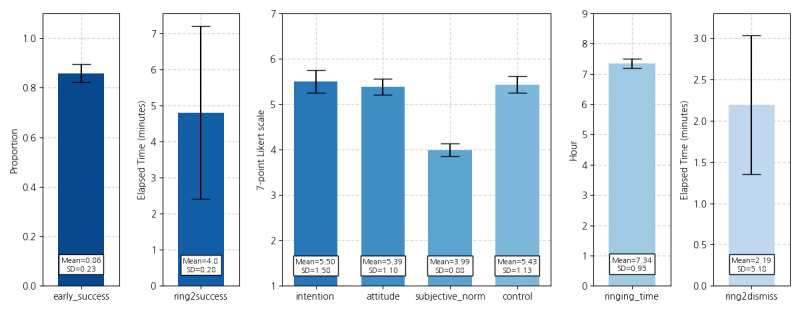
Statistics of the variables for generalized estimation equation analysis.

Figure 6 shows the descriptive statistics of 8 variables. On average, 86% of the participants completed the daily target behavior successfully. The average elapsed time to start the first squat in a successful trial was approximately 4.5 minutes. Both intention and attitude scores were greater than 5 out of 7, indicating that participants tended to be interested in (and had a positive attitude toward) doing squats in the early morning. Participants also seemed to have confidence in doing squats in the early morning. The TPB_subjective_norm score was relatively lower than the others, suggesting that the participants tended not to feel external pressures or expectations about this behavior. Finally, according to ringing_time and ET_ring2dismiss, participants mostly set the wake-up alarm to go off at around 7 AM and spent 2.2 minutes on average, dismissing the alarm after it rang.

Table 1 displays each independent variable’s coefficient in the GEE analysis results. First, we discovered that the alarm dismissal method significantly affected the achievement of early_success. The picture_task was significantly related to the success of morning behavior compared with the non_task (odds ratio [OR] 47.1, 95% CI 2.154-5.554). Similarly, the odds of the math_task group were 3.99 times higher than that of the non_task group (95% CI 0.423-2.347). We also discovered that the odds of success increased more as the user dismissed the alarm earlier (OR 0.652, 95% CI −0.685 to −0.172).

**Table 1:** Results of the generalized estimating equation analysis.

Variables		early_success					ET_ring2success			
		β	SE	*P*	95% CI	β		SE	*P*	95% CI
**Wake-up task**										
	math_task	*1.384*	*0.491*	*.005*	*0.423 to 2.347*	1.282		2.366	.59	−3.355 to 5.921
	picture_task	*3.854*	*0.868*	*<.001*	*2.154 to 5.554*	−*4.052*		*2.288*	*.* *08*	−*8.537 to 0.431*
	non_task (reference category)	N/A^a^	N/A	N/A	N/A	N/A		N/A	N/A	N/A
**Alarm usage**										
	ringing_time	−0.104	0.229	.65	−0.553 to 0.344	−1.473		1.423	.30	−4.263 to 1.316
	ET_ring2dismiss	−*0.428*	*0.131*	*.001*	−*0.685* to *−0.172*	*1.006*		*0.010*	*<.001*	*0.988* to *1.025*
**Theory of planned behavior (TPB)**										
	TPB_intention	*0.605*	*0.321*	*.06*	−*0.024* to *1.234*	0.485		0.812	.55	−1.107 to 2.077
	TPB_attitude	0.408	0.566	.47	−0.702 to 1.518	−3.763		2.420	.12	−8.506 to 0.979
	TPB_subjective_norm	−*1.126*	*0.393*	*.004*	−*1.898* to *−0.356*	*4.143*		*1.911*	*.03*	*0.397* to *7.890*
	TPB_control	−0.218	0.388	.57	−0.979 to 0.542	0.151		1.387	.91	−2.568 to 2.870
**Days**										
	ET_days	−0.013	0.051	.80	−0.114 to 0.087	−0.146		0.288	.61	−0.711 to 0.418

^a^N/A: not applicable.

Next, as shown in Table 1, there were significant factors influencing the elapsed time to success. The subjective norm in TPB was significantly and positively correlated with the elapsed time, indicating that those who feel less pressured tend to begin their squat exercises more quickly. The elapsed time to success tended to be significantly shorter, as the user dismissed the alarm earlier. Furthermore, a marginally significant relationship between picture_task and the elapsed time to success was found, indicating that the user tended to finish the target behavior more quickly when using the picture_task. There was no significant relationship between the other variables and the elapsed time to success.

### Outcome 2: General Practice of Morning Behavior Change by Wake-Up Tasks

#### Quantitative Analysis Results

**Figure 7:**
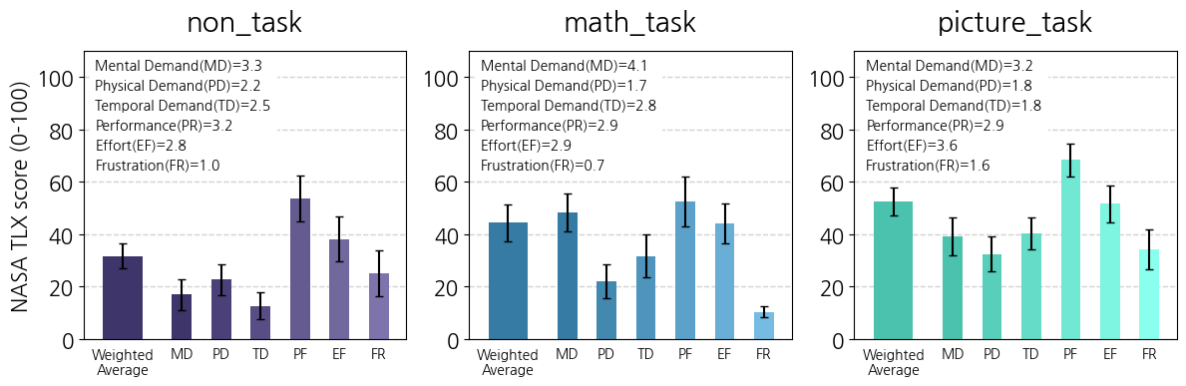
Workloads of alarm dismissal methods (NASA-Task Load Index).

As shown in Figure 7, the total TLX scores of all 3 groups were under 50, indicating an insignificant psychological burden by all methods. The non_task group’s workload was the lowest, followed by the picture_task group and math_task group. We found that the difference in the scores was closely significant and large among the 3 groups’ TLX scores (*F*_2,33_=3.106, *P*=.06, and =0.158), and the posthoc testing results reveal that there was the significant difference between the non_task group and the picture_task group (adjusted *P*=.048 and *d*=1.164). For the 6 subscales, the performance was the highest for all 3 groups, possibly because this study’s tasks involved waking up on time in the morning, which may have satisfied the participants. We also discovered that the type of wake-up tasks appeared to be related to each subscale. For example, the math_task group users tended to perceive a relatively higher mental demand. On the other hand, the picture_task group felt that their task was physically demanding and frustrating.

#### Qualitative Analysis Results

##### Morning Behavior Supports

The participants in this study indicated that they appreciated app functions that supported morning behaviors. First, waking up on time with wake-up tasks was cited as helpful. For example, 1 participant commented, “*Sometimes I just turn off the alarm and lie down again. But, solving a math problem after the alarm fires helped me wake-up easily*” (P34). Another participant, who was a part of the picture_task group, said, “*I had to get up and adjust the position and angle of the target object. I think this makes me wake-up surely, and it became easier to do squats*” (P15). Furthermore, most failure cases were related to waking up late. For example, 1 participant mentioned, “*I turned off the alarm and just tried to sleep a little more. But when I woke up later, over 20 min had passed*” (P7). Second, more than half of the participants (22/36, 61.1%) mentioned the nudging dialog as a useful app function that supported their successful behavior change. It reminded and motivated them to perform the target behavior, with 1 participant saying, *“I often forgot about squats when I just had woke up. But, the dialog message motivated me to start squats each time*” (P18). Similarly, another participant said, “*Even when I finished taking a picture, I sometimes wanted to reject to do squats. But, I felt motivated after seeing the dialog message, and then that button led me to do squats*” (P16). Finally, having the app automatically count the squat reps was cited as helpful by users. One participant said, *“It was convenient that the app counts the number of squats, and I didn’t need to pay much attention to count it*” (P36). Some participants also mentioned that visualizing daily performance encouraged them to keep doing the squats. For example, 1 participant commented, “*I* *was motivated to do harder when I saw the date that I didn’t complete the squats*” (P27).

##### Alarm Setting

We investigated the participants’ alarm setting and their perceived workload. All participants set their alarms for around 7 AM and rarely changed the alarm time. Most participants set the alarm as usual or 5-10 minutes earlier. A participant said, “*I woke up a little earlier to perform the task and squats so that I could keep the same routine as before, such as having breakfast and going to work on time*” (P26). Some participants reported the need to change the alarm time due to a schedule change, but this was not frequent. Next, we analyzed the math_task group’s usage. Considering individual differences in arithmetic operations, we initially allowed participants with the math_task setting to adjust the difficulty level and the number of problems. In the math_task group, 75% (9/12) of the users performed 2 to 3 arithmetic operations that involved adding and subtracting 2 or more numbers of 2 digits. The others preferred more difficult arithmetic operations (ie, multiplication) or more problems. One participant said, “*The example problems were too easy for me, so I increased the difficulty level.*” The picture_task group users usually selected their reference image from between 3 and 5 meters outside their bedroom, such as in their living room or bathroom. The most preferred object for the reference image was a wash basin.

##### Positive Changes in Daily Life

First, several participants noted an improvement in their awakening level. For example, a participant said, *“I have felt more awake in the morning than usual”* (P19). Other participants reported a significant reduction in their half-asleep state in bed. One said, *“I was able to spend less time lying down and dawdling in a bed”* (P29). Second, some participants commented that the app helped them maintain a regular and productive daily routine. These responses were mostly related to waking up on time. By waking up regularly at a target time, participants felt they had more time to spare in the morning and that their daily routines were more consistent. A participant said, *“I liked I could get more time for getting ready for work or taking care of children when I regularly woke up early”* (P25). Another participant stated, *“Usually, I woke up irregularly according to the schedule on that day. But I could live a more consistent life in the past two weeks”* (P32). Finally, some participants provided meaningful comments saying that they started their day in a more positive mood. One participant stated, *“I felt refreshed in the morning as I started a day with a simple exercise”* (P35).

## Discussion

### Principal Results

#### Effectiveness of Wake-Up Tasks for Morning Behavior Change

The results of this study indicate that wake-up tasks can help users effectively use available morning time and maintain their target behavior. Wake-up tasks were significantly and positively associated with target behavior success in the GEE analysis results. The wake-up task groups’ elapsed times to success tended not to be much longer than those of the non_task groups, even though they resulted in a 30-40 second delay in alarm dismissal. When wake-up tasks were completed, the morning time saved relieved the burden of performing the targeted morning behavior, especially for those who had busy mornings (see the comment by R32 for reference). Additionally, participants appreciated the key components of the alarm app developed for this study (ie, alarm, wake-up tasking, and nudging dialog). The mobile alarm helped participants wake up timeously, and the wake-up task after that brought about a winding-up effect by bothering them. Finally, the nudging dialog within the app seemed helpful in getting participants to start their squat exercise routine quickly.

These results are consistent with Fogg’s MAP (motivation, ability, and prompt) theory [13]. The MAP theory outlines 3 factors (motivation, ability, and prompts) to explain behavioral results. In the morning hours, the motivation and ability for the target behavior are likely lower than usual, which can disrupt the behavior’s initiation. Thus, the mobile alarm with wake-up tasks can play a role as an effective prompt for morning behavior by efficiently recovering the normal state of motivation and ability through awakening, warming up, and motivating the user.

Furthermore, our results show a practical use case of inconvenient interaction [55] (or uncomfortable interaction [56,57]). Although most human-computer interaction studies focus on increasing efficiency, some previous studies addressed the need for and the value of inconvenient and uncomfortable interaction. Specifically, Rekimoto and Tsujita [55] suggested that inconvenient design can work in the domain of behavior change. For example, the refrigerator that does not open until the user smiles and the inconvenient microwave that requires the user to perform physical exercises during operation are examples of inconvenient interaction [55]. Unique usage contexts of wake-up tasking are closely related to the principles of inconvenient interaction. Even though the user likely feels inconvenienced by wake-up tasks, these tasks provide immediate and long-term benefits (eg, regular daily routine, positive mood, and physical health) that motivate the user to maintain the behavior.

#### Wake-Up Task Types

This study discovered several differences between the 2 wake-up tasks. First, users using the math_task tended to take a shorter time to dismiss an alarm than those using the picture_task. Similar observations on alarm usage with 2 wake-up tasks were reported in an earlier study based on task-based alarm app usage logs [44]. However, the time between dismissing an alarm and performing the squats tended to be much shorter in the picture_task group than in the math_task group. Similarly, in the GEE analysis, the picture_task group showed a marginally significant relationship with the elapsed time to start behavior, but the math_task group did not. Finally, there were several differences in the workload demands of the 2 tasks. The total workload for the picture_task group was slightly higher than that of the math_task group. Furthermore, although the picture_task group experienced more physical demand and frustration, the math_task group experienced a more demanding mental workload.

These results likely indicate that tasks to elicit behavior change should be designed according to the direction of the anticipated change. Inserting another task between behaviors can be designed in both ways: preventing a particular behavior or preparing for the next. For example, some studies used interruption tasks for behavior change, such as typing digits to limit smartphone use [58], but wake-up tasking prepares the user for more productive morning behavior. The task function and mechanism can differ depending on the behavior goal. Furthermore, the task’s workload amount and type can be essential for task-based behavior change. In this study, the target behavior was a form of physical activity, and participants likely had to move to a location where they could do the required squat exercises. Therefore, the target behavior would be more suitable for those completing a picture_task, which mainly demands a physical workload and similarly moves the users to another place (as they need to capture a target picture). Further studies are necessary to identify the relationship between wake-up tasks and specific target behaviors for a more detailed understanding. It would also be extremely helpful if other types of wake-up tasks (eg, solving a puzzle) and morning behaviors (eg, reading an article) were studied further.

#### Factors Influencing Early Morning Behavior

We also found that common elements for general behavior change (ie, TPB variables) were significantly related to the target behavior success in the morning. For example, TPB_intention was closely significantly and positively associated with early success, and the increase in TPB_subjective_norm was significantly related to decreased behavior performance. However, the results address the need to consider the contextual characteristics of the morning hours because these general variables tended to contribute differently to morning contexts. For example, the magnitude of TPB_intention is smaller than that of the other factors (ie, task usage and alarm usage). Moreover, the correlation direction of TPB_subjective_norm differed from those obtained in other contexts, such as recycling [59] or electronic learning [60]. Therefore, this study is in line with earlier studies that addressed the necessity of considering the contexts of the target behavior [61].

Sleep inertia characterizes the early morning hours. Moreover, people do not usually have much spare time in the early morning because their daily routine begins soon. Therefore, delayed waking up and starting of the target behavior can make users run out of available time and are likely to demotivate them. This study addresses three factors for successful morning behavior change: (1) waking up on time, (2) escaping from sleep inertia, and (3) quickly starting the desired target behavior.

First, it is essential to wake up on time to maintain the anticipated behavior in the early morning. The qualitative study revealed that when the target behavior failed, it was usually due to the user having overslept (see the comment by P7 for reference). Furthermore, most participants with wake-up tasks commented that the tasks helped make them wake up on time and begin the target behavior (see the comment by P15 and P34 for reference). Second, getting ready for the target morning behavior is necessary to escape sleep inertia. The GEE analysis results indicate that the performance of morning behavior decreased as the elapsed time to dismiss became longer, possibly due to oversleeping or a delay in waking up. Even though the participants woke up on time and dismissed their alarms, some remained half-asleep in bed and failed to do the desired target behavior (squats). Waking up late or sleep inertia can negatively affect the success of the target behavior. Finally, having a quick start is another key to performing the target behavior in the morning. The non_task group users tended to dismiss their alarms quickly, but it took a long time to start the target behavior after dismissing the alarm. As a result, the total elapsed time to the target behavior was not too different from that of the other task groups (even marginally lower than the picture_task group). It also had a significantly lower success rate than the other task groups.

Such factors influencing morning behavior can be further extended into a morning routine. Some previous studies for behavior change examined a chain of subsequent behaviors [62,63], and designing behavior can effectively induce other behavior changes [64,65]. Based on this study’s findings, which focused on the beginning of the early morning, we anticipate that designing subsequent morning behaviors as a morning routine may aid in completing productive morning hours, referred to as the miracle morning [1].

### Limitations

This study should be viewed critically because it was conducted at a single site in South Korea for 2 weeks.

This study also used a single target behavior type, squat exercises. In future research, other target behaviors can be studied. For example, reading, regarded as a cognitive activity, could be a good candidate for future morning behavior research. We also received responses from participants who were telecommuting due to COVID-19. However, this is not believed to have significantly influenced the study results because the experiment was conducted in situ by allowing all the participants to set and use alarms at their will.

### Conclusions

Although many people have a positive intention for productive morning behavior, contextual characteristics, such as sleep inertia, make it difficult. This study presents a mechanism for morning behavior change using wake-up tasks in a mobile alarm. Wake-up tasks provide winding-up and nudging effects to help escape from sleep inertia quickly and start the target behavior without subsequent delays. This study’s quantitative and qualitative results confirmed the effectiveness of wake-up tasks in consistently completing the target behavior in the early morning. As a result, this wake-up task mechanism can contribute to extending the existing behavior change research to consider the morning hour context by shedding light on morning hour behaviors.

## Appendix

Multimedia Appendix 1 Results of the presurvey.

## Abbreviations

GEE: generalized estimating equation
MAE: mean absolute error
MAP: motivation, ability, and prompt
NASA-TLX: NASA-Task Load Index
TPB: theory of planned behavior
