# Correction: Using Wake-Up Tasks for Morning Behavior Change: Development and Usability Study

**DOI:** 10.2196/42926

**Published:** 2022-10-03

**Authors:** Kyue Taek Oh, Jisu Ko, Jaemyung Shin, Minsam Ko

**Affiliations:** 1 Department of Human-Computer Interaction Hanyang University Ansan Republic of Korea; 2 Department of Applied Artificial Intelligence Hanyang University Ansan Republic of Korea; 3 Delightroom Seoul Republic of Korea

In “Using Wake-Up Tasks for Morning Behavior Change: Development and Usability Study” (JMIR Form Res 2022;6(9):e39497) the authors noted two errors.

The affiliation of the author Jisu Ko was incorrectly mentioned as the following:

Department of Human-Computer Interaction.

This has been corrected to:

Department of Applied Artificial Intelligence.

Under “Acknowledgments” the original text read:

This work was supported by Institute of Information & communications Technology Planning & Evaluation (IITP) grant funded by the Korea government (Ministry of Science and ICT) (grant RS-2022-00155885, Artificial Intelligence Convergence Innovation Human Resources Development) and Hanyang University ERICA.

It has been replaced by the following:

This work was supported by Institute of Information & communications Technology Planning & Evaluation (IITP) grant funded by the Korea government (Ministry of Science and ICT) (No. RS-2022-00155885, Artificial Intelligence Convergence Innovation Human Resources Development (Hanyang University ERICA)).

The correction will appear in the online version of the paper on the JMIR Publications website on October 3, 2022, together with the publication of this correction notice. Because this was made after submission to PubMed, PubMed Central, and other full-text repositories, the corrected article has also been resubmitted to those repositories.

