# Supplementary material for: Using Wake-Up Tasks for Morning Behavior Change: Development and Usability Study
Source: JMIR Form Res. 2022 Sep 21;6(9):e39497. doi: 10.2196/39497 (PMC9529170; doi:10.2196/39497)
Supplement: Multimedia Appendix 1 [file formative_v6i9e39497_app1.docx]

## Multimedia Appendix 1: Presurvey Results

### I. Demographics

| **Groups** | **# of participants** | **# of females** | **Age** |
| --- | --- | --- | --- |
| *non_task* | 12 | 5 | 27.916 (SD = 8.393) |
| *picture_task* | 12 | 6 | 27.916 (SD = 7.525) |
| *math_task* | 12 | 4 | 26.667 (SD = 7.703) |

### II. Wake-up behavior

| **Question Item** | **Mean (SD)** | | |
| --- | --- | --- | --- |
|  | *non_task* | *picture_task* | *math_task* |
| Please write down the time you usually wake up (target time). | 7.534  (0.059) | 7.333  (0.042) | 7.389  (0.033) |
| How well do you wake up at your target wake-up time?  (7-point Likert scale) | 5.917  (1.311) | 5.250  (0.866) | 5.833  (0.718) |

### III. Responses to questionnaires about theory of planned behavior (7-point Likert scale)

#### A. Intention

| **Question Item** | **Mean (SD)** | | |
| --- | --- | --- | --- |
|  | *non_task* | *picture_task* | *math_task* |
| I intend to exercise every morning right after I wake up. | 5.416 (1.621) | 5.750  (0.965) | 5.416  (2.108) |
| I plan to exercise every morning right after I wake up. | 5.416  (1.621) | 5.083  (1.311) | 5.167  (2.249) |
| I will try to exercise every morning right after I wake up. | 6.083  (1.378) | 5.916  (0.792) | 5.250  (2.261) |
| For me to exercise every morning right after I wake up is interesting | 4.333  (1.669) | 4.416  (1.240) | 4.500  (2.431) |

#### B. Attitude

| **Question Item** | **Mean (SD)** | | |
| --- | --- | --- | --- |
|  | *non_task* | *picture_task* | *math_task* |
| For me to exercise every morning right after I wake up is beneficial | 6.416  (0.792) | 6.500  (0.522) | 6.083  (0.996) |
| For me to exercise every morning right after I wake up is pleasant | 5.333  (1.435) | 5.083  (1.240) | 5.416  (2.193) |
| For me to exercise every morning right after I wake up is valuable | 6.333  (0.887) | 6.250  (0.622) | 6.250  (0.866) |
| For me to exercise every morning right after I wake up is enjoyable | 5.250  (1.422) | 4.500  (1.243) | 4.916  (2.109) |
| For me to exercise every morning right after I wake up is good | 5.916  (1.165) | 5.833  (0.717) | 5.916  (1.240) |
| For me to exercise every morning right after I wake up is satisfied | 5.916  (1.165) | 5.583  (0.900) | 5.750  (1.545) |
| For me to exercise every morning right after I wake up is important | 5.667  (1.435) | 5.583  (1.240) | 4.833  (2.082) |
| For me to exercise every morning right after I wake up is comfortable | 4.833  (1.642) | 4.250  (1.422) | 3.833  (2.209) |

#### C. Perceived subjective norm

| **Question Item** | **Mean (SD)** | | |
| --- | --- | --- | --- |
|  | *non_task* | *picture_task* | *math_task* |
| Most people who are important to me will think it's good to exercise every morning right after I wake up. | 6.250  (0.965) | 6.333  (0.778) | 6.083  (1.165) |
| Most people who are important to me will want to exercise every morning right after I wake up. | 2.083  (1.379) | 2.000  (1.477) | 2.500  (2.111) |
| Most people who are important to me will exercise every morning right after waking up. | 4.583  (1.676) | 4.083  (1.311) | 3.417  (1.505) |
| Many people like me will exercise every morning right after waking up. | 4.000  (1.809) | 3.917  (1.164) | 2.667  (1.723) |

#### D. Perceived behavioral control

| **Question Item** | **Mean (SD)** | | |
| --- | --- | --- | --- |
|  | *non_task* | *picture_task* | *math_task* |
| For me to exercise every morning right after I wake up would be possible | 5.583  (1.443) | 5.833  (0.718) | 5.583  (1.831) |
| I have confidence in exercising right after I wake up every morning. | 5.166  (1.337) | 4.917  (1.311) | 4.750  (2.340) |
| For me to exercise every morning right after I wake up would be easy | 4.666  (1.497) | 4.500  (1.243) | 4.500  (2.195) |
| It is entirely up to me to exercise every morning right after I wake up. | 6.667  (0.492) | 6.500  (0.522) | 6.500  (0.674) |
